# The effects of rice bran supplementation for management of blood lipids: A GRADE-assessed systematic review, dose–response meta-analysis, and meta-regression of randomized controlled trials

**DOI:** 10.1186/s13643-023-02228-y

**Published:** 2023-04-12

**Authors:** Zahra Hariri, Fatemeh Afzalzade, Golbon Sohrab, Saeede Saadati, Zahra Yari

**Affiliations:** 1 Department of Clinical Nutrition and Dietetics, Faculty of Nutrition Sciences and Food Technology, National Nutrition and Food Technology Research Institute, Shahid Beheshti University of Medical Sciences, Tehran, Iran; 2 Department of Clinical Nutrition, School of Nutritional Sciences and Dietetics, Tehran University of Medical Sciences, Tehran, Iran; 3 Department of Medicine, School of Clinical Sciences, Monash University, Melbourne, Australia; 4 Department of Nutrition Research, National Nutrition and Food Technology Research Institute and Faculty of Nutrition Sciences and Food Technology, Shahid Beheshti University of Medical Sciences, Sharake Qods, West Arghavan St. Farahzadi Blvd, Tehran, Iran

**Keywords:** Rice Bran, Lipid Profile, Triglyceride, Cholesterol, Systematic Review, Meta-analysis

## Abstract

**Background:**

We aimed to conduct a systematic review and meta-analysis of randomized controlled trials (RCTs) to investigate the effects of rice bran supplementation on serum lipid profile levels.

**Methods:**

We searched PubMed/Medline, Scopus, ISI Web of Science, and Google Scholar using related keywords. Published RCTs exploring the effects of rice bran consumption on lipid profile were searched up to June 2022. Evidence certainty was assessed on the basis of the Grading of Recommendations, Assessment, Development, and Evaluation (GRADE) approach. The data were pooled using a random-effects model and reported as weighted mean difference (WMD) and 95% confidence interval (CI) for each outcome.

**Results:**

Meta-analysis of eight RCTs (with 11 effect sizes) showed no significant effect of rice bran supplementation on serum levels of triglyceride (WMD: -11.38 mg/dl; 95% CI: -27.73, 4.96; *P* = 0.17), total cholesterol (WMD: -0.68 mg/dl; 95% CI: -7.25, 5.88; *P* = 0.834), low-density lipoprotein cholesterol (WMD: -1.68 mg/dl; 95% CI: -8.46, 5.09; *P* = 0.627) and high-density lipoprotein cholesterol (WMD: 0.16 mg/dl; 95% CI: -1.52, 1.85; *P* = 0.848) compared to control group.

**Conclusion:**

Our meta-analysis suggests that rice bran supplementation has no significant effects on serum levels of lipid profile components. However, larger studies with longer durations and improved methodological quality are needed before firm conclusions can be reached.

**Supplementary Information:**

The online version contains supplementary material available at 10.1186/s13643-023-02228-y.

## Introduction

Dyslipidemia is a multifactorial disorder characterized by a combination (two or more) of increased serum total cholesterol (TC), low-density lipoprotein cholesterol (LDL-C), triglycerides (TG) levels and decreased serum high-density lipoprotein cholesterol (HDL-C) concentrations [1, 2]. The global prevalence of dyslipidemia has increased dramatically over the past 30 years and is among the chronic disease with high mortality rate [3]. Dyslipidemia, as a metabolic abnormality, is recognized as one of the most important risk factors of cardiovascular disease, which accounts for most deaths caused by non-communicable diseases (NCDs) [4].

Pharmacotherapy and lifestyle modifications, especially dietary interventions, are the two main components in optimizing plasma lipid profiles and subsequently reducing the risk of cardiovascular disease [5, 6]. Due to the side effects of drugs, the first line of treatment in dyslipidemia is dietary interventions [6]. Dietary recommendations emphasize the replacement of animal fats with vegetable fats as well as increasing fiber intake. Rice bran (RB) is known as a nutraceutical due to its high fiber content (20–51%), plant sterols and a composition of fatty acids such as oleic acid (38.4%) and linoleic acid (34.4%) [7, 8]. RB also contains a great variety of bioactive phytochemicals, such as γ oryzanol which has a well-defined cholesterol-lowering function [8, 9]. Rice bran contains a wide variety of bioactive compounds with health properties, including amino acids, vitamins and cofactors, and secondary metabolites [10].

The beneficial effects of rice bran, and its derivatives such as rice bran oil, in improving glycemic control [11–13], optimizing lipid profile [14–16], lowering blood pressure [17–19] and weight management [14, 20, 21] have been shown in several studies. An animal study by Zhang et al. [22] showed that fresh rice bran protein can modulate cholesterol metabolism and reduce serum levels of very-low density lipoprotein cholesterol (VLDL-C), LDL-C, TG and hepatic total cholesterol. Qureshi et al. [23–25] also showed the lipid-lowering effects of vitamin E analogues of rice bran in several studies. The lipid-lowering effects of rice bran have also been partly attributed to γ oryzanol, an exclusive rice bran polyphenol [26, 27]. Nevertheless, the results of studies on the effects of rice bran and its components on lipid profile are contradictory and not conclusive. Although two meta-analyses have been published on the effects of rice bran oil on lipid profile [28, 29], meta-analysis has not yet investigated the effects of whole rice bran on lipid profile. Accordingly, we decided to conduct a systematic review and meta-analysis to investigate the effects of RB supplementation on the lipid profile.

## Materials and methods

This systematic review and meta-analysis was registered in PROSPERO before the start of the literature search with registration number CRD42022337982. This study was reported based on Preferred Reporting Items for Systematic Reviews and Meta-Analyses (PRISMA) statement in terms of processing, analyzing, and reporting of the data [30].

### Data sources and search strategies

A systematic literature search was performed in PubMed, Scopus, Web of Science and Google Scholar without specific time frames and language limits, up to June 2022. The purpose of our search was to identify published clinical trials that examined the effects of RB supplementation on lipid profiles (TC, TG, LDL-C, and HDL-C) of adult human.

The following Medical subject headings (MeSH) and non-MeSH terms were used (supplementary): ("rice bran" OR "rice bran powder" OR "rice bran supplement" OR "stabilized rice bran") AND (cholesterol OR "low density lipoprotein" OR LDL OR TC OR "total cholesterol" OR "high density lipoprotein" OR HDL OR "triglyceride" OR TG OR "lipoprotein" OR "lipid profile" OR lipid OR "cardiovascular disease" OR "heart disease" OR "hypercholesterolemia"). In order to complete the search process, a manual screening was performed in article references and review articles, so that no randomized controlled trials (RCTs) were missed.

### Eligibility criteria and study selection

The screening of the titles and abstracts and the further assessment of the full-texts was performed by two independent investigators (Z.H. & F.A.). Studies with the following criteria were included in this meta-analysis: (a) clinical trials (with either parallel or cross-over design), (b) studies which investigated whole rice bran, stabilized rice bran or rice bran powder (c) having a control group (placebo or oral powder similar to rice bran powder) (d) performed in individuals over 18 years old, (e) reported at least one of the following measures: TC and/or TG and/or LDL-C and/or HDL-C.

The exclusion criteria were: (a) animal and in vitro studies (b) studies which investigated the effects of RB concurrently with other interventions, (c) studies which examined only certain components of the RB, such as RB oil, γ oryzanol, ferulic acid, tocols (tocopherol and tocotrienol) or specific amino acids (d) studies which examined defatted RB or RB extract (e) studies without complete information about the outcomes of interest, (f) with less than one-week follow-up, (g) studies without control or placebo group, and (h) studies with other designs except for a clinical trial design.

### Data extraction

The initial screening of articles was done by Z.H. based on the inclusion and exclusion criteria and then double checked by F.A. Any discrepancies in the results were resolved by a third researcher (Z.Y.). In cases where article information was not available, an email was sent to the corresponding author to access the full text of the article. After achieving eligible articles, following data were extracted from each: first author’s name, year of publication, study location, trial duration, gender, mean age and mean body mass index (BMI) of participants, RCT design, the health status of the study population, sample sizes in each group, dose of RB supplementation, and TG (mg/dl), TC (mg/dl), LDL-C (mg/dl) and HDL-C (mg/dl) levels before and after the intervention. This information is presented in Table 1.

**Table 1:** Characteristic of included studies in meta-analysis

studies	Country	Study Design	Participant	Sex	Sample size		Trial Duration (Week)	Means Age		Means BMI		Intervention	
					IG	CG		IG	CG	IG	CG	Rice bran dose (g/d)	Control group
Kestin et al. 1990 (A) [31]	Australia	Cross over (R, DB)	Mildly hypercholesterolemic patients	Male	24	24	4	46.0 ± 10.0	46.0 ± 10.0	25.4 ± 2.0	25.4 ± 2.0	60	Slice of bread or muffin containing 35 g wheat bran
Kestin et al. 1990 (B) [31]	Australia	Cross over (R, DB)	Mildly hypercholesterolemic patients	Male	24	24	4	46.0 ± 10.0	46.0 ± 10.0	25.4 ± 2.0	25.4 ± 2.0	60	Slice of bread or muffin containing 95 g oat bran
Hegsted et al. 1993 [32]	United States	Cross over (R)	Mildly hypercholesterolemic patients	Both	11	11	3	37 ± 10	37 ± 10	26.6 ± 3.4	26.6 ± 3.4	100	Oat bran
Gerhardt et al. 1998 (A) [33]	United States	parallel (R, PC, DB)	Moderately hypercholesterolemic patients	Both	14	13	6	51.7 ± 1.5	51.7 ± 1.5	24.43	24.43	84	Oat bran
Gerhardt et al. 1998 (B) [33]	United States	parallel (R, PC, DB)	Moderately hypercholesterolemic patients	Both	14	17	6	51.7 ± 1.5	51.7 ± 1.5	24.43	24.43	84	Rice starch
Tazakori et al. 2006 [12]	Iran	parallel (PC, DB)	Type 2 Diabetes Mellitus & hypertriglyceridemic	Both	30	30	4	48 ± 10	50 ± 9.3	27.3 ± 3.5	26.2 ± 4	20	White flour without fiber
Matani et al. 2006 [34]	Iran	parallel (R)	Moderately Hypercholesterolemic	Female	10	9	4	51 ± 6	54 ± 7	31 ± 5	28 ± 4	40	wheat bran
Cheng et al. 2010 [13]	Taiwan	parallel (R, PC, DB)	Type 2 Diabetes Mellitus	Both	17	11	12	58.9 ± 10.4	57.7 ± 5.7	25.0 ± 2.2	25.6 ± 2.1	20	milled rice flour
Rondanelli et al. 2011 [35]	Italy	Cross over (R)	Mildly hypercholesterolemic patients	Male	24	24	4	50.33 ± 5.34	50.33 ± 5.34	24.9 ± 1.9	24.9 ± 1.9	30	Beta glucan enriched foods
Borresen et al. 2016 (A) [36]	United States	parallel (R, SB)	Colorectal Cancer Survivors	Both	9	10	4	62 ± 8	59 ± 12	28.7 ± 5.2	28.5 ± 7.9	30	Cooked navy bean powder enriched foods
Borresen et al. 2016 (B) [36]	United States	parallel (R, SB)	Colorectal Cancer Survivors	Both	9	10	4	62 ± 8	64 ± 14	28.7 ± 5.2	27.3 ± 3.3	30	same ingredients as the intervention foods, but did not include NB or RB

*Abbreviations*: *IG* intervention group, *CG* control group, *DB* double-blinded, *SB* single-blinded, *PC* placebo-controlled, *CO* controlled, *R* randomized, *NR* not reported, *F* Female, *M* Male, *NR* not reported

Variables are mean ± SD

### Quality assessment

We classified all studies into 4 groups according to the GRADE guidelines (Grading of Recommendations Assessment, Development, and Evaluation): high, moderate, low, and very low [37].

To evaluate the risk of bias, the Cochrane risk of bias 2.0 tool (RoB 2) per protocol for parallel group randomized trials [38] was used. In this method, risk of bias is evaluated using seven indicators: random sequence generation, allocation concealment, blinding of participants and personnel, blinding of outcome assessment, incomplete outcome data, selective reporting, and other biases. Two researchers independently assessed the risk of bias. Risk of bias was divided into 3 levels: unclear risk (U), low risk (L) and high risk (H) (Additional file 1: Table S1).

### Data synthesis and analysis

In order to evaluate the effects of RB, the mean differences in TG, TC, LDL-C and HDL-C between the intervention and control groups with their standard deviations (SDs) were calculated. The mean change was calculated by following formula: (measure at the end of follow-up in the intervention group—measure at baseline in the intervention group)—(measure at the end of follow-up in the control group—measure at baseline in the control group) [39]. Also, their SDs were calculated as follows: SD = square root [(SD pre-treatment) 2 + (SD post-treatment) 2 -(2R × SD pre-treatment × SD post-treatment)] [39]. In case a study reported the mean change or SD, we considered the same. When standard error of the mean (SEM) was reported instead of SD, we used the following formula to convert it to SD: SEM × √n (n = number of participants in each group). The random-effects model was applied to evaluate the pooled weighted mean difference (WMD) with 95% confidence intervals (CIs). The presence of between-study heterogeneity was assessed by Cochrane’s Q test and I^2^ statistic. I^2^ value > 40% or *P* < 0.05 for the Q-test was characterized as significant between-study heterogeneity [40]. To detect heterogeneity among subgroups, we performed a pre-defined subgroup analysis based on baseline TG, TC, LDL-C and HDL-C, country (USA or non-USA), study design (parallel or cross-over), age (≥ 50 years or less), study duration (≤ 4 weeks or more), RB dose (≥ 60 g/day or less), health status (hypercholesterolemic, non hypercholesterolemic), gender (male, female, or both) and baseline BMI (Normal (18.5–24.9 kg/m^2^), Overweight (25–29.9 kg/m^2^) or Obese (> 30 kg/m^2^)). The potential non-linear effects of RB dose (g/day) and treatment duration (weeks) were investigated using fractional polynomial modeling [41]. Meta-regression analysis was executed to evaluate the association between pooled effect size and RB dose (g/day) and follow-up length (trial duration). A bubble plot was obtained with the size of the "bubble" proportional to the accuracy of the estimate for each of the four factors separately. We also performed the sensitivity analysis method to assess the effect of each study on the overall result, by removing one by one of the studies. Probable publication bias was evaluated by Begg's test [42] (significance point at P < 0.05), Egger's test [43] (and visual funnel plots. All statistical analyzes were performed by STATA software (version 17.0; StatCorp, College Station, TX, USA). In this review *P* < 0.05 was indicated statistically significant.

## Results

### Study selection

**Fig. 1:**
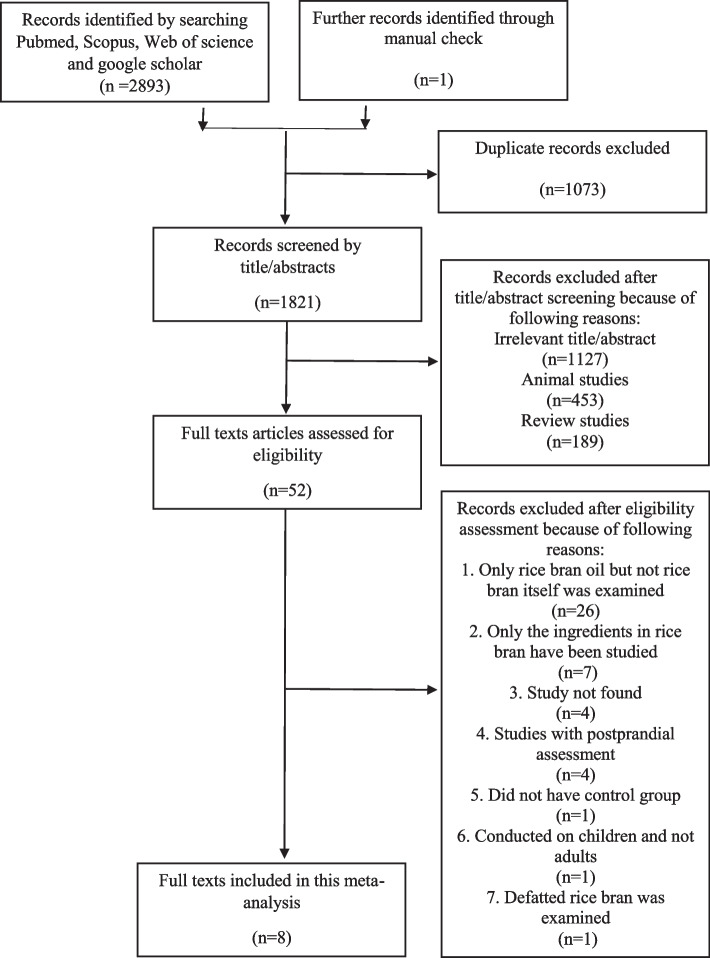
Flowchart of study selection for inclusion trials in the systematic review

Out of 2893 of articles from the database searching and one additional article from reference list checking, 1073 duplicates were removed. Of the remaining 1821 studies, after screening the title and abstract, 1769 were excluded due to lack of relevance (1127) owing to being review articles (189) and being animal studies (453). Fifty-two papers were subjected for thorough full text assessment. Out of that, 44 studies were excluded due to the following reasons: (a) RB oil but not rice bran was examined (*n* = 26), (b) The RB components have been investigated (n = 7) [14, 15, 19, 25, 44–46], (c) Full text paper was not found (*n* = 4) [47–50], (d) Short trial duration (postprandial assessment) (*n* = 3) [51–53], (e) Lack of control group (*n* = 1) [54], (f) Conducted on children (*n* = 1) [55], and (g) Defatted RB was examined (*n* = 1) [56]. Finally, eight eligible RCTs were included in this systematic review and meta-analysis. The PRISMA flow diagram for study selection is shown in Fig. 1.

### Study characteristics

Table 1 summarizes the characteristics of all the included studies. In total, 472 participants (286 cases and 186 controls) with age range between 37 and 62 years old and BMI range between 24.43 and 28.7 kg/m^2^ were recruited.

These RCTs were conducted in Australia [31], United States [32, 33, 36], Iran [12, 34], Taiwan [13] and Italy [35]. Studies were published between 1990 and 2016 and their duration ranged from 3 to 12 weeks. Three of the selected RCTs were designed crossover [31, 32, 35] and the remaining were parallel. The study subjects were patients with hypercholesterolemia [31–35], patients with diabetes [12, 13], and colorectal cancer survivors [36]. Two studies exclusively enrolled men [31, 35], one enrolled only women [34] and the remaining enrolled both sexes [12, 13, 32, 33, 36]. Four studies applied a double blind design [12, 13, 31, 33], one was single-blinded [36] and blindness was not mentioned in the rest [32, 34, 35]. Participants did not take lipid-lowering drugs in none of the studies, although in three studies this was not mentioned [31, 32, 36]. The control group of the studies were as follows: Wheat bran (Kestin et al. (A) [31] and Matani et al. [34]), Oat bran (Kestin et al. (B) [31], Hegsted et al. [32] and Gerhardt (A) [33]), rice starch (Gerhardt et al. (B)[33]), white flour without fiber (Tazakori et al. [12]), milled rice flour (Cheng et al. [13]), beta-glucan enriched foods (Rondanelli et al. [35]), navy bean powder (Borresen et al. (A) [36]) and same ingredients as the intervention foods but not include RB (Borresen et al. (B) [36]).

### Quality assessment

In terms of random sequence generation, two studies had low risk of bias [31, 35], five studies had unclear risk [13, 32–34, 36] and one had high risk [12]. Allocation concealment was unclear in all studies, with the exception of two studies which had a low risk of bias [33, 36]. All the trials had a low risk of bias regarding selective outcome reporting. Three studies were performed double blinded so considered low risk [13, 31, 33], one was single blinded (high risk) [36] and the others did not mention blinding (unclear risk) [34, 35]. Outcome assessor blinding was ruled out in two studies (high risk) [13, 36], but was unclear in the other studies (unclear risk). None of the studies had incomplete outcome data or other sources of bias, so they were considered low risk in both aspects. After evaluation of studies based on the above seven criteria, if a study met more than two high-risk indicators, it was considered generally high-risk, if it met two indicators, it was considered moderate risk and if it met less than two indicators, it was considered low risk.

As a whole, all studies had a low risk of general bias, except for two studies that had a moderate risk of general bias [34, 36].

### Meta-analysis results

The effects of RB supplementation on TG, LDL-C, HDL-C and TC were evaluated by eight studies (with 11 effect sizes) involving 369 participants (186 cases and 183 controls).

### Effect of RB supplementation on TG concentrations

Eleven effect sizes, including a total of 369 participants (186 intervention and 183 control subjects) assessed the effect of RB supplementation on circulating TG levels. The overall meta-analysis reported that RB supplementation does not significantly change serum TG levels (WMD: -11.38 mg/dl; 95% CI: -27.73, 4.96; P = 0.17) (Additional file 1: Figure S2.A). Also a significant degree of heterogeneity was found (I^2^ = 79.5%, *P* < 0.001). Subgroup analysis showed a significant decrease in TG in studies involving both sexes, overweight and obese individuals, and 50 years’ old participants or younger (Table 2).

**Table 2:** Subgroup analyses of rice bran supplementation on lipid profile in adults

				heterogeneity		
	Number of effect sizes	WMD (95%CI)	*P*-value	P heterogeneity	I^2^	P between sub-groups
**Subgroup analyses of rice bran supplementation on triglyceride (TG)**						
Overall effect	11	-11.38 (-27.73, 4.96)	0.17	< 0.001	79.5%	
Baseline TG (mg/dl)						
< 150	7	-8.73 (-24.81, 7.34)	0.287	0.001	72.3%	0.949
≥ 150	4	-10.44 (-60.94, 40.05)	0.685	< 0.001	87.6%	
Country						
USA	5	-10.22 (-28.71, 8.27)	0.279	0.083	51.5%	0.906
non-USA	6	-12.38 (-43.05, 18.29)	0.429	< 0.001	87.5%	
Type of study						
Parallel	7	-11.23 (-43.54, 21.08)	0.496	< 0.001	81.5%	0.868
Cross-over	4	-8.05 (-27.18, 11.07)	0.409	0.001	80.7%	
Age (year)						
< 50	4	-27.58 (-49.01, -6.15)	**0.012**	< 0.001	85.5%	0.048
≥ 50	7	2.59 (-18.36, 23.56)	0.808	0.043	53.9%	
Trial duration (week)						
≤ 4	8	-11.56 (-30.55, 7.41)	0.232	< 0.001	83.5%	0.947
> 4	3	-10.12 (-48.39, 28.14)	0.604	0.063	63.9%	
Intervention dose (g/day)						
< 60	6	-11.02 (-48.50, 26.44)	0.564	< 0.001	87.2%	0.979
≥ 60	5	-11.57 (-26.35, 3.19)	0.124	0.042	59.7%	
Health status						
Hypercholesterolemic	7	-0.00 (-18.39, 18.38)	0.999	< 0.001	77.4%	0.098
non-hypercholesterolemic	4	-33.48 (-68.61, 1.64)	0.062	0.003	78.7%	
Sex						
Both sexes	3	-22.80 (-43.27, -2.34)	**0.029**	< 0.001	75.5%	0.023
Male	7	-0.48 (-18.46, 17.49)	0.958	0.123	52.2%	
Female	1	-11.38 (-27.73, 4.96)	0.050			
Baseline BMI (kg/m^2^)						
Normal (18.5–24.9)	3	14.34 (-4.18, 32.87)	0.129	0.465	0.0%	0.001
Overweight (25–29.9)	7	-24.73 (-41.02, 08.45)	**0.003**	< 0.001	75.4%	
Obese (> 30)	1	62.000 (-0.08, 124.08)	**0.050**			
**Subgroup analyses of rice bran supplementation on total cholesterol (TC)**						
Overall effect	11	-0.68(-7.25, 5.88)	0.834	< 0.001	79.5%	
Baseline TC (mg/dl)						
< 200	0	-	-	-	-	-
≥ 200	11	-0.68 (-7.25, 5.88)	0.834	< 0.001	92.3%	
Country						
USA	5	-2.18 (-9.89, 5.52)	0.579	0.006	72.0%	0.699
non-USA	6	0.86 (-12.54, 14.27)	0.899	< 0.001	84.0%	
Type of study						
Parallel	7	-4.26 (-15.62, 7.09)	0.462	< 0.001	79.9%	0.250
Cross-over	4	4.52 (-5.23, 14.29)	0.363	0.001	82.8%	
Age (year)						
< 50	4	-1.32 (-5.21, 2.57)	0.506	0.301	18.0%	0.936
≥ 50	7	-0.77 (-13.66, 12.12)	0.907	< 0.001	86.1%	
Trial duration (week)						
≤ 4	8	2.32 (-3.71, 8.36)	0.451	0.002	69.2%	0.318
> 4	3	-12.17 (-39.97, 15.63)	0.391	< 0.001	91.5%	
Intervention dose (g/day)						
< 60	6	-0.94(-13.57, 11.68)	0.924	< 0.001	83.7%	0.949
≥ 60	5	-0.43(-9.44, 8.57)	0.883	0.002	76.6%	
Health status						
Hypercholesterolemic	7	4.12(-4.73, 12.97)	0.362	< 0.001	83.1%	0.063
non-Hypercholesterolemic	4	-9.09(-19.81, 1.63)	0.097	0.030	66.5%	
Sex						
Both sexes	7	-5.75(-13.33, 1.826)	0.137	< 0.001	77.1%	0.020
Male	3	7.53(-3.68, 18.75)	0.188	0.054	65.8%	
Female	1	25.00(0.26, 49.73)	0.048			
Baseline BMI (kg/m^2^)						
Normal (18.5–24.9)	3	3.23(-17.37, 23.84)	0.758	< 0.001	90.3%	0.072
Overweight (25–29.9)	7	-3.97(-9.89, 1.95)	0.189	0.018	60.7%	
Obese (> 30)	1	25.00(0.26, 49.73)	0.048			
**Subgroup analyses of rice bran supplementation on low density lipoprotein cholesterol (LDL-C)**						
Overall effect	11	-1.68(-8.46, 5.09)	0.627	< 0.001	81.2%	
Baseline LDL-C(mg/dl)						
< 130	3	-13.60 (-38.34, 11.13)	0.281	< 0.001	89.6%	0.245
≥ 130	8	1.62(-5.30, 8.55)	0.646	< 0.001	76.8%	
Country						
USA	5	-2.58 (-9.07, 3.90)	0.435	0.035	61.3%	0.792
non-USA	6	-0.29 (-16.03, 15.45)	0.971	< 0.001	88.1%	
Type of study						
Parallel	7	-5.17 (-18.71, 8.37)	0.454	< 0.001	85.2%	0.274
Cross-over	4	3.36 (-3.76, 10.48)	0.355	0.024	68.3%	
Age (year)						
< 50	4	-0.64 (-6.34, 5.04)	0.823	0.130	46.8%	0.861
≥ 50	7	-2.00 (-16.04, 12.04)	0.780	< 0.001	87.3%	
Trial duration (week)						
≤ 4	8	2.78 (-3.12, 8.69)	0.356	0.005	65.5%	0.132
> 4	3	-17.78 (-43.88, 8.31)	0.182	< 0.001	91.6%	
Intervention dose (g/day)						
< 60	6	-1.73 (-17.59, 14.11)	0.830	< 0.001	87.1%	0.942
≥ 60	5	-1.09 (-8.29, 6.09)	0.765	0.007	71.9%	
Health status						
Hypercholesterolemic	7	3.14 (-4.47, 10.77)	0.419	< 0.001	79.0%	0.107
non-Hypercholesterolemic	4	-12.33 (-29.56, 4.88)	0.160	< 0.001	84.3%	
Sex						
Both sexes	7	-8.11 (-16.79, 0.55)	0.067	< 0.001	82.3%	0.001
Male	3	6.55 (-1.14, 14.24)	0.095	0.221	33.7%	
Female	1	36.00 (11.62, 60.37)	**0.004**			
Baseline BMI (kg/m^2^)						
Normal (18.5–24.9)	3	-0.46 (-18.07, 17.14)	0.959	0.001	86.7%	0.007
Overweight (25–29.9)	7	-4.91 (-12.80, 2.97)	0.222	< 0.001	78.9%	
Obese (> 30)	1	36.00 (11.62, 60.37)	**0.004**			
**Subgroup analyses of rice bran supplementation on high density lipoprotein cholesterol (HDL-C)**						
Overall effect	11	0.16 (-1.52, 1.85)	0.848	0.005	60.3%	
Baseline HDL-C (mg/dl)						
< 40	1	-3.00 (-12.53, 6.53)	0.538			0.508
≥ 40	10	0.27 (-1.47, 2.03)	0.758	0.003	64.1%	
Country						
USA	5	-1.50 (-1.90, -1.10)	** < 0.001**	0.599	0.0%	0.138
non-USA	6	0.96 (-2.27, 4.21)	0.558	0.006	69.7%	
Type of study						
Parallel	7	0.82 (-2.25, 3.89)	0.601	0.006	67.0%	0.145
Cross-over	4	-1.48 (-1.88, -1.08)	** < 0.001**	0.451	0.0%	
Age (year)						
< 50	4	1.65 (-2.17, 5.49)	0.397	< 0.001	86.6%	0.214
≥ 50	7	0.16 (-1.52, 1.85)	0.248	0.855	0.0%	
Trial duration (week)						
≤ 4	8	0.35 (-1.87, 2.58)	0.756	0.002	69.4%	0.534
> 4	3	-0.70 (-3.19, 1.78)	0.578	0.342	6.8%	
Intervention dose (g/day)						
< 60	6	0.31 (-3.17, 3.79)	0.830	0.861	71.7%	0.566
≥ 60	5	-0.77 (-2.08, 0.52)	0.243	0.243	18.8%	
Health status						
Hypercholesterolemic	7	-1.45(-1.85, -1.06)	** < 0.001**	0.535	0.0%	0.297
non-Hypercholesterolemic	4	0.90(-3.51, 5.33)	0.688	0.001	82.5%	
Sex						
Both sexes	7	0.41(-1.92, 2.76)	0.726	0.001	73.6%	0.788
Male	3	0.39(-1.98, 2.78)	0.743	0.921	0.0%	
Female	1	-3.00(-12.53, 6.53)	0.538			
Baseline BMI (kg/m^2^)						
Normal (18.5–24.9)	3	1.05(-2.52, 4.64)	0.563	0.793	0.0%	0.724
Overweight (25–29.9)	7	0.17(-1.88, 2.22)	0.870	0.001	73.7%	
Obese (> 30)	1	-3.00(-12.53, 6.53)	0.538			

*Abbreviations*: *CI* confidence interval, *WMD* weighted mean differences, *TG* Triglyceride, *TC* Total cholesterol, *LDL* low density lipoprotein, *HDL* high density lipoprotein

### Effect of RB supplementation on TC concentrations

Overall, 11 arms of included clinical trials (186 intervention and 183 control subjects) investigated the effect of RB supplementation on TC concentration, and pooled effect size showed a non-significant decreased serum TC concentration (WMD: -0.68 mg/dl; 95% CI: -7.25, 5.88; *P* = 0.834) with a significant heterogeneity between studies (I^2^ = 79.5%, *P* < 0.001) (Additional file 1: Figure S2.B). Furthermore, performing subgroup analyses, we did not find any significant effect of RB intake on TC levels among all the subgroups (Table 2).

### Effect of RB supplementation on LDL-C concentrations

In total, 11 effect sizes with a sample size of 369 participants were included in the analysis. Combining these effect sizes, a significant reduction was not seen in serum concentrations of LDL-C following RB supplementation (WMD: -1.68 mg/dl; 95% CI: -8.46, 5.09; *P* = 0.627) (Additional file 1: Figure S2.C). In addition, the degree of heterogeneity was significant (I^2^ = 81.2%, *P* < 0.001). In addition, the subgroup analysis revealed that in the study which exclusively enrolled obese women, the reduction in LDL-C following RB supplementation was significant (Table 2).

### Effect of RB supplementation on HDL-C concentrations

The meta-analysis of 11 effect sizes involving 369 individuals revealed no significant change in HDL-C levels after RB intervention (WMD: 0.16 mg/dl; 95% CI: -1.52, 1.85; *P* = 0.848) compared with control group (Additional file 1: Figure S2.D). The amount of heterogeneity was also notable among the studies (I^2^ = 60.3%*, P* = 0.005). Based on the analysis, in studies conducted in the United States, in cross-over studies and those with patients with hypercholesterolemia the intervention group experienced a lower increase or even a decrease in HDL-C compared to the control group (Table 2).

### Sensitivity analysis

In order to evaluate the contribution of each study to the final result of this meta-analysis, we removed each study in turn and assessed the results without them. As a result of this analysis, the overall effect size was not influenced by a single study except for HDL-C that the overall effect was changed significantly with the omission of Tazakori et al. study [12] (WMD: -1.46 mg/dl, 95%CI: -1.85, -1.07).

### Publication bias

According to Eager's test, Begg's test and visual inspection of funnel plots, no publication bias was detected in studies evaluating the effect of RB supplementation on TG (*P* = 0.204, SE: 1.02, CI: -0.91—3.73, Egger’s test) (*P* = 0.276, Begg's test), TC (*P* = 0.844, SE: 0.94, CI: -1.94—2.32, Egger’s test) (*P* = 0.436 Begg's test), LDL-C (*P* = 0.981, SE: 0.88, CI: -1.99–2.03, Egger’s test) (*p* = 0.276 Begg's test) or HDL-C (*P* = 0.081, SE: 0.50, CI: -0.15–2.13, Egger’s test) (*P* = 0.436 Begg's test) (Additional file 1: Figure S3 A-D).

### Non-linear dose–response analysis between dose and duration of RB supplementation and lipid profile

According to dose–response analysis, RB dose variety could significantly alter TG (*r* = -911.19, SE: 359.22, CI: -1739.55 – 82.82, P _nonlinearity_ = 0.035), TC (*r* = 265.27, SE:55.22, CI: 137.92– 392.63, P _nonlinearity_ = 0.001), and LDL-C (*r* = -547.14, SE:55.22, CI: 137.92– 392.63, P _nonlinearity_ = 0.023) but did not alter HDL-C (*r* = 34.70, SE:35.64, CI: -47.48 – 116.89, P _nonlinearity_ = 0.359) significantly (Additional file 1: Figure S4 A-D). Furthermore, dose–response analysis based on duration showed that although supplementation with RB significantly altered LDL-C (*r* = 1573.83, SE: 415.94, CI: 614.66–2532.99, P _nonlinearity_ = 0.005), it did not significantly change TG (*r* = 70.85, SE: 57.90, CI: -62.68 – 204.38, P _nonlinearity_ = 0.256), and HDL-C (*r* = -216.14, SE: 163.16, CI: -592.41– 160.12, P _nonlinearity_ = 0.222). The changes in TC levels were close to the significant level (*r* = 1019.33, SE: 452.79, CI: -24.75– 2063.42, P _nonlinearity_ = 0.054) (Additional file 1: Figure 5 A-D).

### Meta-regression analysis

Meta-regression analysis was performed to investigate the possible relationship between RB dose, study duration and lipid profile changes. Based on this analysis, no significant association was detected between RB supplementation dose and changes in TG (Slope = 0.17, Intercept = 53.82, SE: 0.310, CI: -0.53 – 0.87, P _linearity_ = 0.595), TC (Slope = 0.16, Intercept = 51.12, SE:0.60, CI: -1.21 – 1.53, P _linearity =_ 0.798), LDL-C (Slope = 0.15, Intercept = 51.39, SE:0.50, CI: -0.98 – 1.29, P _linearity =_ 0.763) or HDL-C (Slope = -0.44, Intercept = 50.90, SE:3.03 CI: -7.31 – 6.42, P _linearity =_ 0.887) levels (Additional file 1: Figure 6 A-D). Similarly, the relationship between study duration and changes in TG (Slope = 0.00, Intercept = 3.66, SE:0.14 CI: -0.32 – 0.32, P _linearity =_ 0.981), TC (Slope = -0.05, Intercept: 3.27, SE:0.14 CI = -0.37 – 0.27, P _linearity =_ 0.721), LDL-C (Slope = -0.08, Intercept = 3.03, SE:0.13 CI: -0.39 – 0.22, P _linearity =_ 0.533), and HDL-C (Slope = -0.17, Intercept = 5.13, SE:0.35 CI: -0.97 – 0.63, P _linearity =_ 0.640) levels were not significant (Additional file 1: Figure 7 A-D).

### Grading of evidence

The grading of evidence is presented in Additional file 1: Table S2. The quality of studies evaluating the effect of RB supplementation on TG, TC, LDL-C and HDL-C were deemed to be low due to their heterogeneity percentage between studies and their insignificancy.

## Discussion

For the first time in this systematic review and meta-analysis, we assessed the effects of RB supplementation on lipid profile changes among human adults. After analyzing the eight studies (with 11 effect sizes), we concluded that RB supplementation has no statistically significant effect on improving the serum levels of TG, TC, LDL-C, and HDL-C compared to control group. However, based on the subgroup analysis, the effect of RB on TG levels was significant in studies involving both men and women, as well as in studies in which participants were older than 50 years old. This significance was mainly attributed to the study of Hegsted et al. [32]. In this study, the effect of 100 g of stabilized RB supplementation in patients with hypercholesterolemia was investigated and since 100 g was the highest dose of RB supplementation among the studies, this significant difference can be attributed to the high dose of RB in this study [32]. Furthermore, based on a non-linear dose–response analysis, RB dose variety could significantly change TG, TC and LDL-C levels, but the variation of the study duration only caused a significant change in LDL-C concentration. Meta-regression analysis was performed as well to detect the possible linear association between dose and duration and changes in lipid profile, but did not show a significant association.

The results of this meta-analysis are inconsistent with the results of some of the RCTs included in this review. Tazkari et al. investigated the effectiveness of RB supplementation on lipid profile changes in patients with diabetes and reported that RB supplementation could significantly reduce TG levels and increase HDL-C levels [12]. In another study in patients with diabetes, it was found that supplementation with stabilized RB was able to significantly reduce TC and LDL-C concentrations [13].

Notably, two meta-analyses have been investigated the effect of RB oil on lipid profile changes [28, 29]. Jolfaie et al. which included 11 RCTs found that RB oil supplementation could reduce the risk of cardiovascular disease through reducing TC and LDL-C levels [28]. In another meta-analysis, Pourrajab et al. showed a significant effect of RB oil on TG reduction in addition to TC and LDL-C [29].

In addition to oil, rice bran also contains fiber and protein, which is considered in the present study. The results of previous study indicated that the effects of rice bran fiber concentrates on lowering TC and LDL-C in patients with diabetes was significantly higher than rice bran water soluble concentrates and stabilized rice bran [11]. There are about 21 g of dietary fiber per 100 g of RB, while the same amount of oat bran contains 15.4 g of fiber [57, 58]. 90% of RB dietary fiber is insoluble which includes cellulose, hemicellulose and arabinoxylans, and 10% is soluble fiber, which is mainly pectin and β-glucan [59–61]. Soluble fiber can lower blood cholesterol through following mechanisms: (a) binding to bile acids thus acting as bile acid sequestrate, (b) increasing short chain fatty acids (SCFAs) production and decreasing hepatic cholesterol production, (c) slowing down the absorption of carbohydrates, (d) reducing insulin secretion and thus reducing cholesterol production [62]. It has also been shown that RB soluble fiber could down-regulate the expression of genes involved in lipogenesis and significantly reduce TG, TL and LDL-C levels [63].

Although in general the cholesterol-lowering effect of soluble fiber is greater than that of insoluble fiber [64], mechanisms for improving lipid profile have also been described for insoluble fiber. By increasing the fecal bulk (bulking effect), insoluble fiber reduces the intestinal transit time and thus reduces fat absorption [65]. Another possible mechanism of insoluble fiber is to induce a long-term satiety [66]. It has also been shown that RB protein can exhibit lipid-lowering effects by preventing the binding of cholesterol to bile acids, thereby lowering serum cholesterol and increasing fat excretion [67].

A distinct feature of rice bran is its high oil content (≈ 20.8%) compared to other bran, including wheat (≈ 7.03%) and oat (≈ 4.25%) bran [58]. Despite the mentioned benefits for the defatted rice bran, a comparison of defatted RB and RB oil showed significant lipid lowering effects of RB oil [68]. Lipid-lowering effects of RB seem to be attributed to γ oryzanol, high amounts of vitamin E and excellent fatty acid profile [8, 57]. γ oryzanol can lower cholesterol through a variety of mechanisms, including: inhibition of cholesterol-esterase [69, 70] and increased fecal excretion of cholesterol and bile acids [27, 71]. Two mechanisms has been also proposed for lipid-lowering effects of tocopherol: antioxidant activity against cholesterol oxidation [72] and 3-hydroxy-3methylglutaryl-coenzyme A (HMG-CoA) reductase inhibiting [73–76].

It seems that the dose of RB oil supplemented in RCTs was higher than the oil content of rice bran. Therefore, studies investigating the effects of RB oil have reported a significant improvement in lipid profile changes.

There are some strengths in the present systematic review and meta-analysis. This is the first meta-analysis to evaluate the effects of RB supplementation on lipid profile in human adults. It has also relatively acceptable number of studies and large sample sizes. There was no time or language limitation while searching the databases. Moreover, to discover the cause of heterogeneity, a subgroup analysis was performed. All participants showed baseline TC > 200 mg/dl, which is considered as a borderline for hypercholesterolemia according to National Cholesterol Education Program (NCEP) III guidelines [77]. Sensitivity analysis was also performed for TG, TC and LDL-C parameters which showed that no study distinctly affected the overall result. No publication bias was discovered among studies according to Eager's test, Begg's test and visual inspection of funnel plots.

Despite these strengths, following limitations should be taken into account while interpreting the results. There was an insufficient number of RCTs, and most of the RCTs had relatively small sample sizes. The presence of only one female single-sex group in the subgroup analysis based on gender, reduces the significance. Some RCTs were open-labeled, which can affect the outcome. The degree of heterogeneity was also significant between studies, and this may be due to the limited number of studies and their small sample sizes. Besides, the dose of the study also ranged from 20 to 100 g/day, and this variation made the comparison more complicated. As a result, these limitations suggest that more placebo controlled randomized clinical trials with larger sample sizes are needed to determine the true effect of RB supplementation on lipid profile.

## Conclusion

The present systematic review and meta-analysis disclosed that supplementation with rice bran did not show significant effects on serum levels of TG, TC, LDL-C and HDL-C. Given the existing contradictions, for more accurate and reliable conclusion on the effects of rice bran on serum lipid profiles, more clinical trials with larger sample sizes and different doses and durations are needed.

## Supplementary Information

**Additional file 1:** **Table S1. **Risk of bias assessment.** Table S2. **GRADE profile of rice bran supplementation for lipid profile. **Figure S2.A. **Forest plot detailing weighted mean difference and 95% confidence intervals (CIs) for the effect of rice bran supplementation on triglycerides (TG). **Figure S2.B. **Forest plot detailing weighted mean difference and 95% confidence intervals (CIs) for the effect of rice bran supplementation on total cholesterol (TC). **Figure S2.C. **Forest plot detailing weighted mean difference and 95% confidence intervals (CIs) for the effect of rice bran supplementation on low density lipoprotein cholesterol (LDL-C). **Figure S2.D.** Forest plot detailing weighted mean difference and 95% confidence intervals (CIs) for the effect of rice bran supplementation on high density lipoprotein cholesterol (HDL-C). **Figure S3. **Funnel plot for the effect of rice bran supplementation on A) TG; B) TC; C) LDL-C and D) HDL-C. Abbreviations: TG: triglycerides; TC: total cholesterol; LDL-C: low-density lipoprotein cholesterol; HDL-C: high-density lipoprotein cholesterol. **Figure S4. **Non-linear dose-response relations between dose of rice bran supplementation (g/day) and absolute mean differences in A) TG; B) TC; C) LDL-C and D) HDL-C. Abbreviations: TG: triglycerides; TC: total cholesterol; LDL-C: low-density lipoprotein cholesterol; HDL-C: high-density lipoprotein cholesterol.** Figure S5.  **Non-linear dose-response relations between duration of intervention (week) and absolute mean differences in A) TG; B) TC C) LDL-C and D) HDL-C. Abbreviations: TG: triglycerides; TC: total cholesterol; LDL-C: low-density lipoprotein cholesterol; HDL-C: high-density lipoprotein cholesterol.** Figure S6.  **Bubble plots of the association between dose of rice bran (g/day) and weighted mean difference of A) TG; B) TC; C) LDL-C and D) HDL-C. The size of the bubbles is proportional to the accuracy of the estimate. Abbreviations: TG: triglycerides; TC: total cholesterol; LDL-C: low-density lipoprotein cholesterol; HDL-C: high-density lipoprotein cholesterol. **Figure S7. **Bubble plots of the association between duration of intervention and weighted mean difference of A) TG; B) TC C) LDL-C and B) HDL-C. The size of the bubbles is proportional to the accuracy of the estimate. Abbreviations: TG: triglycerides; TC: total cholesterol; LDL-C: low-density lipoprotein cholesterol; HDL-C: high-density lipoprotein cholesterol. **Search strategy.**

## Data Availability

All data generated or analyzed during this study are included in this manuscript.

## Abbreviation

RCTs: Randomized controlled trials
TC: Total cholesterol
LDL-C: Low-density lipoprotein cholesterol
TG: Triglycerides
HDL-C: High-density lipoprotein cholesterol
NCDs: Non-communicable diseases
RB: Rice bran
VLDL-C: Very-low density lipoprotein cholesterol
PRISMA: Preferred Reporting Items for Systematic Reviews and Meta-Analyses
MeSH: Medical subject headings
BMI: Body mass index
GRADE: Grading of Recommendations Assessment, Development, and Evaluation
SDs: Standard deviations
SEM: Standard error of the mean
WMD: Weighted mean difference
CIs: Confidence intervals
SCFAs: Short chain fatty acids
HMG-CoA: 3-Hydroxy-3methylglutaryl-coenzyme A
NCEP: National Cholesterol Education Program
