# Supplementary material for: The effects of rice bran supplementation for management of blood lipids: A GRADE-assessed systematic review, dose–response meta-analysis, and meta-regression of randomized controlled trials
Source: Syst Rev. 2023 Apr 12;12:65. doi: 10.1186/s13643-023-02228-y (PMC10091523; doi:10.1186/s13643-023-02228-y)
Supplement: Supplementary file 1 — Additional file 1: Table S1. Risk of bias assessment. Table S2. GRADE profile of rice bran supplementation for lipid profile. Figure S2.A. Forest plot detailing weighted mean difference and 95% confidence intervals (CIs) for the effect of rice bran supplementation on triglycerides (TG). Figure S2.B. Forest plot detailing weighted mean difference and 95% confidence intervals (CIs) for the effect of rice bran supplementation on total cholesterol (TC). Figure S2.C. Forest plot detailing weighted mean difference and 95% confidence intervals (CIs) for the effect of rice bran supplementation on low density lipoprotein cholesterol (LDL-C). Figure S2.D. Forest plot detailing weighted mean difference and 95% confidence intervals (CIs) for the effect of rice bran supplementation on high density lipoprotein cholesterol (HDL-C). Figure S3. Funnel plot for the effect of rice bran supplementation on A) TG; B) TC; C) LDL-C and D) HDL-C. Abbreviations: TG: triglycerides; TC: total cholesterol; LDL-C: low-density lipoprotein cholesterol; HDL-C: high-density lipoprotein cholesterol. Figure S4. Non-linear dose-response relations between dose of rice bran supplementation (g/day) and absolute mean differences in A) TG; B) TC; C) LDL-C and D) HDL-C. Abbreviations: TG: triglycerides; TC: total cholesterol; LDL-C: low-density lipoprotein cholesterol; HDL-C: high-density lipoprotein cholesterol. Figure S5. Non-linear dose-response relations between duration of intervention (week) and absolute mean differences in A) TG; B) TC C) LDL-C and D) HDL-C. Abbreviations: TG: triglycerides; TC: total cholesterol; LDL-C: low-density lipoprotein cholesterol; HDL-C: high-density lipoprotein cholesterol. Figure S6. Bubble plots of the association between dose of rice bran (g/day) and weighted mean difference of A) TG; B) TC; C) LDL-C and D) HDL-C. The size of the bubbles is proportional to the accuracy of the estimate. Abbreviations: TG: triglycerides; TC: total cholesterol; LDL-C: low-density lipoprotei [file 13643_2023_2228_MOESM1_ESM.docx]

| **Table 1.** Risk of bias assessment | | | | | | | | |
| --- | --- | --- | --- | --- | --- | --- | --- | --- |
| **Study** | **Random sequence generation** | **Allocation concealment** | **Selective reporting** | **Other sources of bias** | **Blinding (participants and personnel)** | **Blinding (outcome assessment)** | **Incomplete outcome data** | **General risk of bias** |
| Kestin et al. 1990 (A) | L | U | L | L | L | U | L | Low |
| Kestin et al. 1990 (B) | L | U | L | L | L | U | L | Low |
| Hegsted et al. 1993 | U | U | L | L | H | U | L | Low |
| Gerhardt et al. 1998 (A) | U | L | L | L | L | U | L | Low |
| Gerhardt et al. 1998 (B) | U | L | L | L | L | U | L | Low |
| Tazakori et al. 2006 | H | U | L | L | L | U | L | Low |
| Matani et al. 2006 | U | U | L | L | U | U | L | Moderate |
| Cheng et al. 2010 | U | U | L | L | L | H | L | Low |
| Rondanelli et al. 2011 | L | U | L | L | U | U | L | Low |
| Borresen et al. 2016 (A) | U | L | L | L | H | H | L | Moderate |
| Borresen et al. 2016 (B) | U | L | L | L | H | H | L | Moderate |
| Abbreviations. L, low risk of bias; H, high risk of bias; U, unclear risk of bias.  General Low risk < 2 high risk  General moderate risk = 2 high risk  General high risk < 2 high risk | | | | | | | | |

| **Table 2.** GRADE profile of rice bran supplementation for lipid profile. | | | | | | | | |
| --- | --- | --- | --- | --- | --- | --- | --- | --- |
| **Quality assessment** | | | | | | **Summary of findings** | | **Quality**  **of evidence** |
| **Outcomes** | **Risk of bias** | **Inconsistency** | **Indirectness** | **Imprecision** | **Publication Bias** | **Number of intervention / control** | **WMD (95%CI)** |  |
| TG | No serious limitations | Very serious limitation ^a^ | No serious limitation | Serious limitation ^e^ | No serious limitation | 186/183 | -11.38(-27.73, 4.96) | ⊕⊕◯◯  Low |
| TC | No serious limitations | Very serious limitation ^b^ | No serious limitation | Serious limitation ^e^ | No serious limitation | 186/183 | -0.68 (-7.25, 5.88) | ⊕⊕◯◯  Low |
| LDL-C | No serious limitations | Very serious limitation ^c^ | No serious limitation | Serious limitation ^e^ | No serious limitation | 186/183 | -1.68 (-8.46, 5.09) | ⊕⊕◯◯  Low |
| HDL-C | No serious limitations | serious limitation ^d^ | No serious limitation | Serious limitation ^e^ | No serious limitation | 186/183 | 0.16 (-1.52, 1.85) | ⊕⊕◯◯  Low |
| ^a^ The test for heterogeneity is significant, and the I^2^ is low, 79.5%. ^b^ The test for heterogeneity is significant, and the I^2^ is low, 79.5%. ^c^ The test for heterogeneity is significant, and the I^2^ is low, 81.2%. ^d^ The test for heterogeneity is significant, and the I^2^ is low, 60.3%. ^e^ Confidence intervals contain zero. Based on the number of limitations, the quality of outcomes is divided into four categories: high (⊕⊕⊕⊕), moderate (⊕⊕⊕◯), low (⊕⊕◯◯) and very low (⊕◯◯◯) quality.  Abbreviations: TG: triglycerides; TC: total cholesterol; LDL-C: low-density lipoprotein cholesterol; HDL-C: high-density lipoprotein cholesterol. | | | | | | | | |

**Figure 2.A** Forest plot detailing weighted mean difference and 95% confidence intervals (CIs) for the effect of rice bran supplementation on triglycerides (TG).

**Figure 2.B** Forest plot detailing weighted mean difference and 95% confidence intervals (CIs) for the effect of rice bran supplementation on total cholesterol (TC).

**Figure 2.C** Forest plot detailing weighted mean difference and 95% confidence intervals (CIs) for the effect of rice bran supplementation on low density lipoprotein cholesterol (LDL-C).

**Figure 2.D** Forest plot detailing weighted mean difference and 95% confidence intervals (CIs) for the effect of rice bran supplementation on high density lipoprotein cholesterol (HDL-C).

| **A**  **** | **B**  **** |
| --- | --- |
| **C**  **** | **D**  **** |
| **Figure 3.** Funnel plot for the effect of rice bran supplementation on A) TG; B) TC; C) LDL-C and D) HDL-C.  Abbreviations: TG: triglycerides; TC: total cholesterol; LDL-C: low-density lipoprotein cholesterol; HDL-C: high-density lipoprotein cholesterol. | |

| **A**  **** | | **B**  **** |
| --- | --- | --- |
| **C**  **** | | **D**  **** |
| **Figure 4.** Non-linear dose-response relations between dose of rice bran supplementation (g/day) and absolute mean differences in A) TG; B) TC; C) LDL-C and D) HDL-C.  Abbreviations: TG: triglycerides; TC: total cholesterol; LDL-C: low-density lipoprotein cholesterol; HDL-C: high-density lipoprotein cholesterol. | | |
| **A**  **** | **B**  **** | |
| **C**  **** | **D**  **** | |
| **Figure 5.** Non-linear dose-response relations between duration of intervention (week) and absolute mean differences in A) TG; B) TC C) LDL-C and D) HDL-C.  Abbreviations: TG: triglycerides; TC: total cholesterol; LDL-C: low-density lipoprotein cholesterol; HDL-C: high-density lipoprotein cholesterol. | | |

| **A**  **** | **B**  **** |
| --- | --- |
| **C**  **** | **D**  **** |
| **Figure 6.** Bubble plots of the association between dose of rice bran (g/day) and weighted mean difference of A) TG; B) TC; C) LDL-C and D) HDL-C. The size of the bubbles is proportional to the accuracy of the estimate.  Abbreviations: TG: triglycerides; TC: total cholesterol; LDL-C: low-density lipoprotein cholesterol; HDL-C: high-density lipoprotein cholesterol. | |

| **A**  **** | **B**  **** |
| --- | --- |
| **C**  **** | **D**  **** |
| **Figure 7.** Bubble plots of the association between duration of intervention and weighted mean difference of A) TG; B) TC C) LDL-C and B) HDL-C. The size of the bubbles is proportional to the accuracy of the estimate.  Abbreviations: TG: triglycerides; TC: total cholesterol; LDL-C: low-density lipoprotein cholesterol; HDL-C: high-density lipoprotein cholesterol. | |

**Search strategy:**

**PubMed**: ("rice bran"[Title/Abstract] OR "rice bran powder"[Title/Abstract] OR "rice bran supplement"[Title/Abstract] OR "Stabilized Rice Bran"[Title/Abstract]) AND (cholesterol[Title/Abstract] OR "low density lipoprotein"[Title/Abstract] OR LDL[Title/Abstract] OR TC[Title/Abstract] OR "total cholesterol"[Title/Abstract] OR "high density lipoprotein"[Title/Abstract] OR HDL[Title/Abstract] OR "triglyceride"[Title/Abstract] OR TG[Title/Abstract] OR "lipoprotein"[Title/Abstract] OR "lipid profile"[Title/Abstract] OR Lipid[Title/Abstract] OR "cardiovascular disease"[Title/Abstract] OR "heart disease"[Title/Abstract] OR "hypercholesterolemia"[Title/Abstract])

**Scopus**: ( TITLE-ABS-KEY ( "rice bran" OR "rice bran powder" OR "rice bran supplement" OR "Stabilized Rice Bran" ) AND TITLE-ABS-KEY ( cholesterol OR "low density lipoprotein" OR ldl OR tc OR "total cholesterol" OR "high density lipoprotein" OR hdl OR "triglyceride" OR tg OR "lipoprotein" OR "lipid profile" OR lipid OR "cardiovascular disease" OR "heart disease" OR "hypercholesterolemia" ) )

**Web of science**: TOPIC: ("rice bran" OR "rice bran powder" OR "rice bran supplement" OR "Stabilized Rice Bran") AND TOPIC: (cholesterol OR "low density lipoprotein" OR LDL OR TC OR "total cholesterol" OR "high density lipoprotein" OR HDL OR "triglyceride" OR TG OR "lipoprotein" OR "lipid profile" OR Lipid OR "cardiovascular disease" OR "heart disease" OR "hypercholesterolemia")
